# High-resolution surface faulting from the 1983 Idaho Lost River Fault M_w_ 6.9 earthquake and previous events

**DOI:** 10.1038/s41597-021-00838-6

**Published:** 2021-02-26

**Authors:** Simone Bello, Chelsea P. Scott, Federica Ferrarini, Francesco Brozzetti, Tyler Scott, Daniele Cirillo, Rita de Nardis, J Ramón Arrowsmith, Giusy Lavecchia

**Affiliations:** 1 DiSPUTer - Dipartimento di Scienze Psicologiche, della Salute e del Territorio, Università G. d’Annunzio Chieti-Pescara, Chieti, Italy; 2 CRUST - Centro InteRUniversitario per l’analisi Sismotettonica Tridimensionale, Chieti, Italy; 3 School of Earth and Space Exploration – Arizona State University, Arizona, USA

**Keywords:** Tectonics, Structural geology

## Abstract

We present high-resolution mapping and surface faulting measurements along the Lost River fault (Idaho-USA), a normal fault activated in the 1983 (M_w_ 6.9) earthquake. The earthquake ruptured ~35 km of the fault with a maximum throw of ~3 m. From new 5 to 30 cm-pixel resolution topography collected by an Unmanned Aerial Vehicle, we produce the most comprehensive dataset of systematically measured vertical separations from ~37 km of fault length activated by the 1983 and prehistoric earthquakes. We provide Digital Elevation Models, orthophotographs, and three tables of: (i) 757 surface rupture traces, (ii) 1295 serial topographic profiles spaced 25 m apart that indicate rupture zone width and (iii) 2053 vertical separation measurements, each with additional textual and numerical fields. Our novel dataset supports advancing scientific knowledge about this fault system, refining scaling laws of intra-continental faults, comparing to other earthquakes to better understand faulting processes, and contributing to global probabilistic hazard approaches. Our methodology can be applied to other fault zones with high-resolution topographic data.

## Abstract (summary)

**Table:** 

Measurement(s)	earthquake surface ruptures • quaternary fault scarps • earthquake surface rupture Vertical Separation • quaternary fault scarps rupture Vertical Separation • rupture zone widths • active geological fault
Technology Type(s)	Geographic Information System • computational modeling technique
Sample Characteristic - Environment	tectonic earthquake • intracontinental extensional normal fault
Sample Characteristic - Location	United States of America • State of Idaho • Lost River Range

Machine-accessible metadata file describing the reported data: 10.6084/m9.figshare.13198259

## Background & Summary

In the past 40 years, numerous moderate-to-large intra-continental extensional earthquakes (M_w_ 6–7) have generated complex surface ruptures along primary and secondary synthetic and antithetic splay faults. In-depth studies of these systems contribute to understanding earthquake recurrence rates, surface rupture processes, fault displacement hazard, and the tectonic significance of these fault systems at late-Quaternary timescales.

**Fig. 1:**
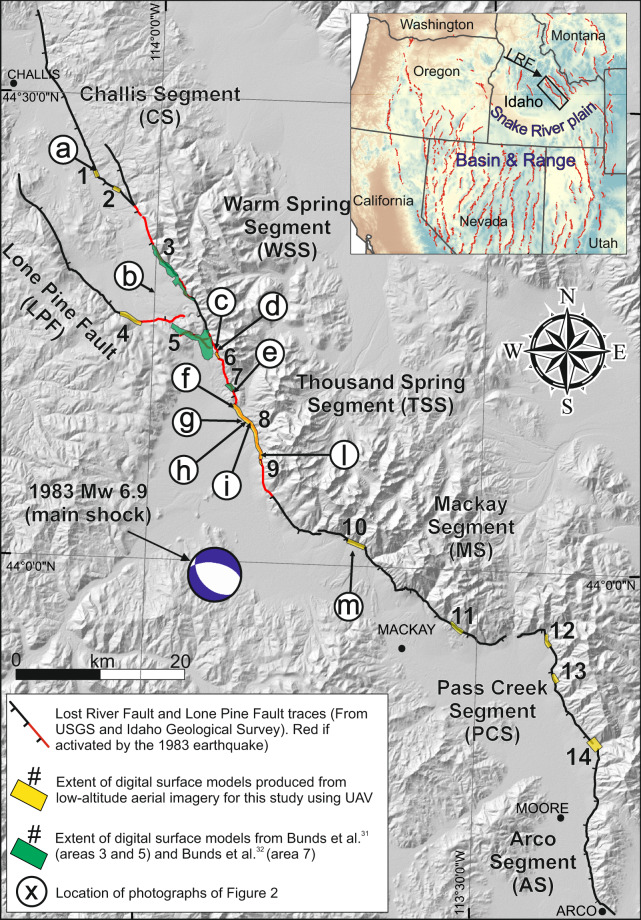
Lost River Fault (LRF) and nearby study areas. Black lines show the LRF with ticks on downthrown side. Red lines show the portion of the LRF activated by the 1983 Borah Peak earthquake. Faults are from USGS. Numbered polygons show areas mapped with high-resolution topography^30^, (yellow=this work; green=Bunds *et al*.^31,32^). Circled letters (a - m) correspond to the photographs in Fig. 2. Inset map shows location of the LRF in the Basin and Range extensional intra-continental tectonic province of the western USA. 1983 Borah Peak main shock focal mechanism is from Doser and Smith^3^.

In 1983, the Borah Peak earthquake (M_w_ 6.9, hereinafter referred to as 1983Eq), one of the largest and most recent normal-faulting earthquakes in the United States, ruptured ~35 km of the ~130-km-long Lost River Fault (LRF) in southeastern Idaho (Fig. 1). The LRF is in the northernmost portion of the Basin and Range Province^1^, strikes ~N25°W and dips ~75°SW. The LRF and the 1983Eq have been the focus of seminal investigations. Multiple studies constrained the fault geometry at depth, the seismic sequence, and tectonic strain from shallow seismic lines, seismological data and GPS velocities^2–8^, highlighting the nucleation of the rupture at a depth of ~16 km at the southern tip of the activated fault (Fig. 1) with subsequent northwestward propagation. Geodetic data suggested a planar high-angle source fault^9–11^. Some studies characterized the surface and depth deformation pattern dividing the fault with boundaries and complexities in six ~SW-dipping active normal segments: Challis, Warm Springs, Thousand Springs, Mackay, Pass Creek, and Arco^12–16^. The Thousand Springs and the southern Warm Springs segments were activated in 1983 with a normal-oblique rupture mechanism (Fig. 1). In particular, Crone *et al*.^13^, mapped the surface ruptures over the ~37 km ruptured fault and measured the vertical (Supplementary Figure 1) and the strike-slip components, highlighting a ~17% left-lateral component of the total slip. Others constrained the timing of multiple prehistoric surface faulting events^17–22^ from Quaternary geology, paleo-seismological trenching and radionuclide dating. DuRoss *et al*.^23^ reexamined the surface deformation produced by the 1983Eq, showing that structural-geological complexities present along the fault guided the coseismic deformation pattern along its northern 16 km and providing new mapping and vertical separation measurements (Supplementary Figure 1).

High-resolution surface deformation datasets from normal faults are limited to a few recent earthquakes^24–28^. Baize *et al*.^29^ unifies this datatype from literature studies in a consistent database. Our objective was to collect and systematically analyze vertical separations (VS) along the LRF using newly acquired high-resolution topography. We define VS as the vertical distance between the intersection of a vertical plane at the fault and lines projected along the hanging wall (HW) and footwall (FW) surfaces assumed to be continuous prior to their displacement.

In spring 2019, we imaged ~21 km along-strike of the LRF (Fig. 1) using a Phantom 4-Pro Unmanned Aerial Vehicle (UAV) flying at 70–120 m elevation above ground level. Images were geolocated with on-board GNSS (Global Navigation Satellite System) and differential dGNSS Ground Control Points. We processed the images in Agisoft Metashape photogrammetric modeling software (versions 1.6.0) to produce high-resolution Digital Elevation Models and orthophotos^30^. We also used data from Bunds *et al*.^31,32^ (~16 km along-strike) to create hillshades. From the above datasets, we mapped the observable 1983 coseismic surface ruptures and Quaternary fault scarps (hereinafter referred to as respectively CoRs and Qfs). For quality control, we assigned each trace an Outcrop Quality Ranking (OQR) on a 1-to-4 scale, based on the faulting evidence in the high-resolution image (1 is best). We created an interactive MATLAB (www.mathworks.com) algorithm that we used to make 2053 VS measurements along 1295 fault-perpendicular topographic profiles^33^ with a 25 m spacing. We assigned a Measure Quality Ranking (MQR) to each VS measurement considering the vegetation, the angle between the HW and FW, and the fault position. Two geoscientists independently analyzed 10% of the profiles to access subjectivity. We provide the mapped traces as shapefiles, three tables that provide geometric information on the CoRs and Qfs of the areas shown in Fig. 1, VS measurements, methodology, topographic profiles, and quality parameters stored in *Pangaea*^34^.

This database provides new high-resolution information on recent-ground-rupturing earthquakes along the LRF, a major active extensional fault. Our data are critical for informing paleoseismic, tectonic geomorphology and structural geologic investigations of the LRF, as well as for characterizing probabilistic fault displacement hazard analysis^29^, the effect of geometric discontinuities on rupture extent, and slip-length scaling in large earthquakes^35–38^. Our methodology advances systematic approaches for measuring fault scarp profiles from the growing archive of high-resolution topography.

## Methods

### Preparation and field campaign

In ArcMap©, we compiled scientific results about prehistoric and the 1983Eq along the LRF^13,14,19–23,39–45^. We used satellite imagery to define potential field sites, focusing on accessibility, vegetation covering, landownership, topographic altitude, and “no-fly zone” areas. In April 2019, we acquired ~19,000 photographs using two DJI Phantom 4 Pro drones that flew at ~70–120 m altitude above ground level. In total, we covered ~21 km along fault strike of the LRF with an average imaging width of ~417 m, as shown in Fig. 1. We describe the characteristics of the areas in Table 1.

**Table 1:** Characteristics of the high-resolution topographic data areas.

Area ID	Area name	Fault	Segment	Area (km^2^)	Length along strike (m)	Mean width (m)	Number of topographic profiles	First T. Pr.	Last T. Pr.	Source
1	Hole-in-Rock Creek	LRF	CHS	~0.35	~845	~420	33	38	70	This work
2	Lime Creek	LRF	CHS	~0.39	~943	~400	37	1	37	This work
3	Warm Springs Segment*	LRF	WSS	~4.5	~8680	~450	335	71	405	Bunds *et al*.^31^
4	Broken Wagon Creek	LPF	-	~1.2	~2800	~390	111	406	516	This work
5	Northern Thousand Springs*	LRF	TSS	~7.6	~6000	~1000	251	517	767	Bunds *et al*.^31^
6	Dickey Peak*	LRF	TSS	~0.16	~740	~210	32	768	799	This work
7	Poison Spring*	LRF	TSS	~0.61	~1000	~550	47	800	846	Bunds *et al*.^32^
8	Thousand Springs*	LRF	TSS	~3.18	~7721	~500	308	847	1154	This work
9	Southern Thousand Springs*	LRF	TSS	~0.13	~570	~210	25	1155	1179	This work
10	Petes Creek	LRF	MS	~1	~2117	~470	82	1180	1261	This work
11	Mahogany Gulch	LRF	MS	~0.79	~1710	~430	NaN	NaN	NaN	This work
12	Jepson Canyon	LRF	PCS	~0.48	~1487	~340	34	1262	1295	This work
13	Maddock canyon	LRF	PCS	~0.44	~1020	~470	NaN	NaN	NaN	This work
14	Ramshorn Canyon	LRF	PCS	~0.91	~1300	~750	NaN	NaN	NaN	This work

Areas ID in Fig. 1. Area names with apex asterisk are areas with 1983 coseismic surface ruptures; Key: LRF = Lost River Fault; LPF = Lone Pine Fault; CHS = Challis Segment; TSS = Thousand Springs Segment; MS = Mackay Segment; PCS = Pass Creek Segment. First T. Pr. and Last T. Pr. refer to the ID of the topographic profile reported in the Topographic Profiles Dataset^34^.

**Fig. 2:**
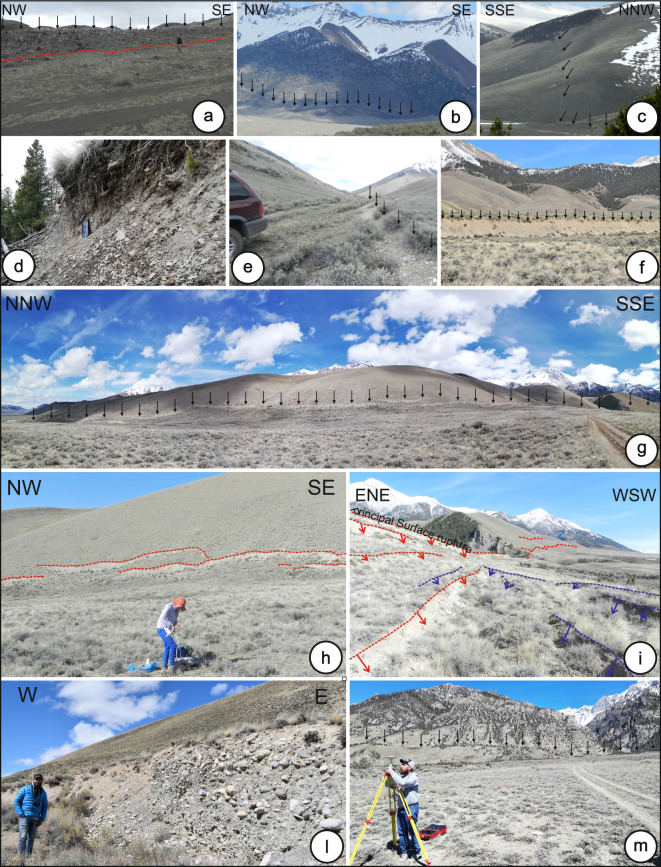
1983 Coseismic surface ruptures (CoRs) and Quaternary fault scarps (Qfs) along the LRF (location in Fig. 1). (**a**) Example of a Qfs along the Challis segment (Area 1); black arrows highlight the scarp top; photograph taken looking NE. (**b**) CoRs along the Warm Springs Segment (Area 3); photograph taken looking NE. (**c**) CoRs at Area 5; photograph taken looking NW. (**d**) CoRs along the Thousand Springs Segment (Area 6); photograph taken looking NNW; Apple iPad Pro for scale. (**e**) CoRs along the Thousand Springs Segment (area 7). (**f**) CoRs along the Thousand Springs Segment (area 8). (**g**) CoRs along the Thousand Springs Segment (Area 8). (**h**) A geoscientist setting up a UAV along the Thousand Springs Segment with CoRs, (red dashed lines), observed from SW towards NE. (**i**) CoRs (red and blue dashed lines highlight synthetic and antithetic CoRs, respectively) along the Thousand Springs Segment; photograph taken looking SSE. (**l**) A CoR free face well-preserved due to indurated conglomerate; photograph taken looking N. (**m**) A geoscientist preparing the dGNSS station along the Mackay segment (Area 10). The Qfs are highlighted by variation in vegetation along the fault.

Images have geolocation information from onboard GNSS with a 10 m accuracy. We used reduce error along the Thousand Springs Segment and Mackay Segment (areas 8 and 10), from ground control points (GCP) measured with a dGNSS. We placed the ~1-m-square black and white vinyl GCP targets on both sides of the fault. Along the Thousand Spring (~4.35 km^2^ of imagery) and Mackay Segment (~1 km^2^), we used 17 (~4 GCP/ km^2^) and 12 GCPs, respectively. We measured GCP locations with a GPS1200 base station (Fig. 2m), an RX1200 rover with an INTUICOM antenna, and a Leica AX1202GG tripod. The GCP position accuracy is ~0.02 m in the horizontal and vertical directions for area 10 and ~1.2 m and ~2.8 m, respectively, for area 8. We corrected the station locations using the National Geodetic Survey’s Online Positioning User Service (Opus^46^; http://www.ngs.noaa.gov/OPUS/) and reprojected positions into WGS84 UTM zone 12 N.

### Processing of aerial images and mapping

**Fig. 3:**
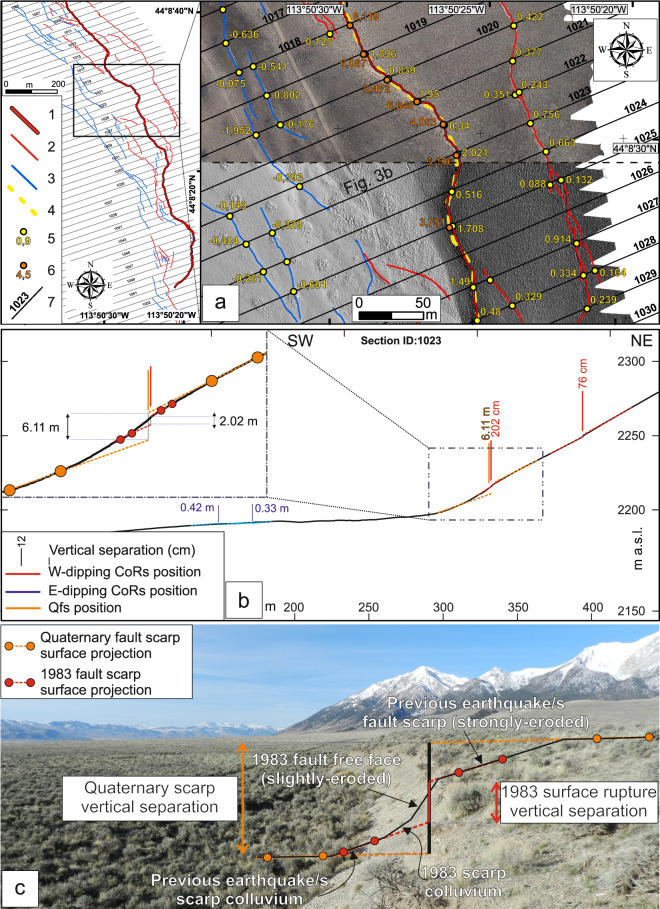
Analysis of fault scarp profiles from high-resolution topography. (**a)** Key: 1 = Principal surface coseismic rupture; 2 = Distributed synthetic surface coseismic rupture; 3 = Distributed antithetic surface coseismic rupture; 4 = Synthetic Quaternary fault scarp; 5 = 1983 surface rupture vertical separation; 6 = Quaternary fault scarp vertical separation; 7 = Topographic profile trace. Example of orthomosaic (upper half) and hillshade map (lower half) from a portion (location in left inset) of the study area 8. We used these images to map principal 1983 coseismic surface ruptures (P-CoRs - thick red line), distributed 1983 coseismic surface ruptures (D-CoRs - thin red or blue lines) and Qfs (orange dashed line). We measure VS for CoRs (1983 coseismic surface ruptures; yellow points) and Qfs (Quaternary fault scarp; orange points) from the topographic profiles (black lines). The circles are exactly where VS was measured. (**b)** Example topographic profile (trace in a) used to measure VS. The zoom shows the linear surface projections for measuring VS of a CoR (red) and Qfs (orange). Vertical lines show the fault location. (**c)** Illustration of VS measurement on a photograph at Double Spring Pass road, along the Thousand Springs segment (after DuRoss *et al*.^23^). The vertical back line shows the fault location.

We manually selected the UAV images and eliminated those with a low quality, blurred, or acquired by mistake (for example, takeoff and landing photos). We processed the selected photographs with Agisoft Metashape image-based photogrammetric modeling software (Version 1.6.0) to produce dense point clouds, orthomosaics and digital elevation models (DEMs). Figure 3a shows an example of Orthomosaic and hillshade produced from a DEM^47–53^. The initial alignment was highest quality. Dense point clouds, mesh, and texture were made with high-quality settings. The DEM and orthophotos were exported with the default recommended resolution (2–30 cm/pix). The DEMs were then used to build slope maps, hillshade maps, curvature maps and aspect maps on ArcMap (ESRI ArcMap© 10.7)^30^. We also used DEMs and orthomosaics hosted by OpenTopography (https://opentopography.org) produced by Bunds *et al*.^31,32^ (areas 3, 5 and 7 of Fig. 1).

The DEMs and orthomosaics were used for mapping in ArcMap© at a fixed scale 1:400, also taking into consideration the maps produced by previous authors^13,23^. We mapped keeping the same continuous line for each clearly visible trace on the 1:400 scale of our DEMs and orthomosaics. The accuracy of the mapping is therefore reproducible at this scale. Figure 2 presents representative photos of the surface faulting. Figure 3a shows a detail of the map (hillshade and orthomosaic) where we mapped CoRs and Qfs. During the digital mapping, we assigned three attributes to fault traces: an identification number, the type of trace (Principal/distributed CoR or Qfs) and the dip direction (W-dip or E-dip). We assigned the “Type” attribute to the CoRs on the basis of four parameters considered: the dip-direction, the rupture length, the along-strike continuity and the amount of VS. In areas where only one CoR was visible, we *ex officio* assigned the Principal CoR attribute. Where instead there were more parallel CoRs, we assigned the Principal CoR attribute to those synthetic structures with greater continuity, length and/or VS. We have assigned the Distributed CoR attribute to all the remaining CoRs and, even in this case *ex officio*, to all the antithetic CoRs. These attributes were used in the profile analysis described below. An outcrop-quality ranking (OQR) was also assigned to each trace. The OQR consists of a 1 to 4 ranking (ascending quality; OQR 1 = very high, OQR 4 = very low), assigned based on the evidence of the trace on the high-resolution image (i.e., outcrop quality).

### Sequential analysis of fault-crossing topographic profiles

The main challenge was to investigate the topography along ~37 km of fault and efficiently measure vertical separation (VS). We developed a MATLAB algorithm that we used to systematically measure VS along 1295 topographic profiles^33^. The ~150 surface offset measurements from Crone *et al*.^13^ document a minor left-lateral slip component of the 1983Eq ruptures. With our methodology we only measured the vertical component of the fault displacement. Following DuRoss *et al*.^23^, we ignored the ~17% of the moment released as left-lateral slip, considering it to have minimal influence. Our VS measurements can therefore be considered appropriate for future normal-fault surface-rupture processes studies.

The inputs were the DEMs and the mapped fault traces. We tiled the DEMs using the “Split Raster” tool from ArcMap© (see the guide provided^33^). The topographic profiles that were generated from the DEMs have a 25-m spacing and with elevations every 20 cm from a 30 cm moving window. The 25-m spacing ensured that we made at least one VS measurement for almost every CoRs, even for relatively short ones. The 2 m averaging window minimized the impact of the topography. The profiles are orientated perpendicular to the average rupture strike in the individual areas (Fig. 1). Due to the complex pattern of ruptures characterized by distributed CoRs with variable strike, the profiles are not always perpendicular to the rupture traces. The vertical component (VS) of the displacement is not affected by the variation of the angle of the topographic profile only if there is no slope variation in the along-strike direction. In other cases, it can affect the measurements (discussion in the technical validation section).

A graphical interface shows vertical lines along the topographic profile (red and blue for west- and for east-dipping faults, respectively) from the traces mapped in ArcMap©. To measure VS, we marked two points along each of the HW and FW to be used for the respective surface projections. While choosing the four points for the linear surface projections, we considered the small bushes that form the vegetation. While we did not classify vegetation, we selected bare ground points while measuring VS and avoided vegetation easily identifiable on orthomosaics, DEMs and topographic profiles. The possibility to change the lighting direction (to the hillshades made from DEMs) helped in this process.

A fifth point associates the measurement with the trace ID. A sixth point indicates the position where the fault intersects the topography. We consider the scarp morphology degradation and accumulation factors to estimate position^18,54–60^, which often corresponds to the steepest part of the scarp face.

Figure 3b shows a topographic profile with CoRs and Qfs and the points that we used to build the linear surface projections for the VS. Figure 3c is a photograph of the Double Springs Pass road area showing a natural example of the geometry used to interpret the 1295 topographic profiles.

As shown in Figs. 2 and 3, CoRs are distinguishable from Qfs in the DEMs and orthomosaics. To measure VS, we picked four points within a few meters of the CoRs and within tens of meters of the Qfs. As established in the literature^13,23^ and from our mapping, the 1983Eq produced a complex pattern of synthetic and antithetic coseismic ruptures, forming grabens and horsts. These structures vary in width substantially along the fault trace. We aimed to distinguish individual synthetic and antithetic CoRs while measuring VS. When this was not always possible (for example when the CoRs were within ~3–4 m of each other) because there was insufficient length for robust linear surface projections, we picked the four points in the first suitable position and added up the values of VS. For example in the case of a graben, we measured the principal rupture from far-field points. The graphical interface closes after the sixth point and reopens immediately showing the FW and HW linear surface projections, and the VS in centimeters. After seeing the projected lines, a seventh point confirm the fault position. Finally, it is decided whether to keep the measurement or, if there is a mistake, delete it and redo the interpretation.

Following a decision to keep the measurement, the object (CoR or Qfs) and a measure-quality ranking (MQR) are saved in a MATLAB structure file. The MQR has a 1-to-4 value based on three parameters: the presence of vegetation, the angle between the linear surface projections at the HW and FW, and the trace position. A MQR = 1 (high-quality) indicates absent or minimal vegetation, a low angle between the linear surface projections (<30°) and a clear trace position. When the ground surface is completely covered with vegetation, there is a high-angle between linear surface projections (>30°), or factors such as high erosion make identifying the trace challenging, we assign MQR = 4 (low-quality).

In addition, the MATLAB structure file includes the horizontal position of each clicked point and the VS. The graphical output from MATLAB is saved as a MATLAB figure and .EPS file. The compilation of the database derives from the storage of these information which are then exported in a .txt file. We subsequently opened these .txt files in Microsoft Excel where we homogenized and screened them and where we added other important textual and numerical information not originally saved in MATLAB.

We provide the data organized in a simple database that is usable by other researchers. We compiled three tables: (1) Traces of mapped CoRs and Qfs, (2) topographic profiles, and (3) measurements acquired on topographic profiles. To make features uniquely identifiable, we assign a progressive ID to each individual trace, topographic profile and VS measurement.

**Fig. 4:**
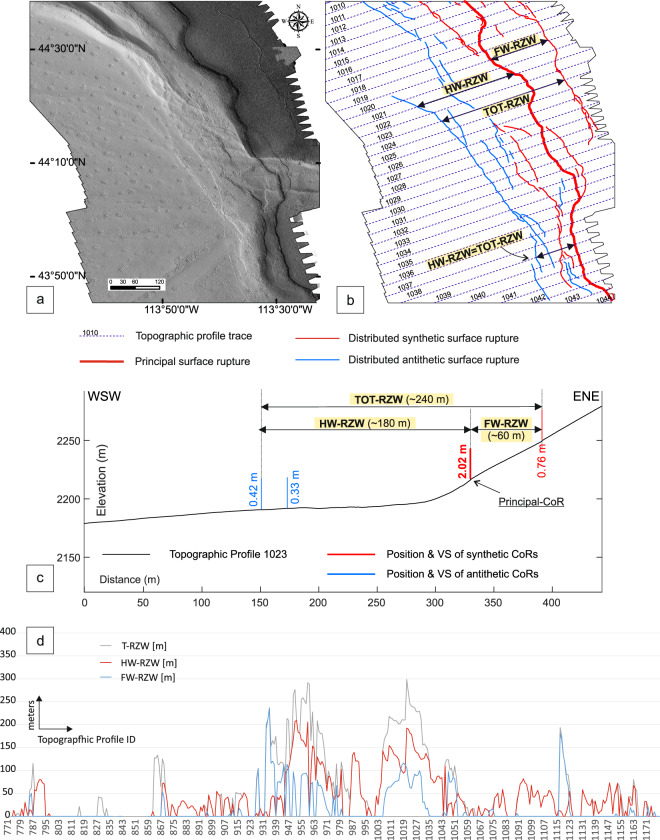
Rupture Zone Widths (RZWs). (**a)** Topographic hillshade map in area 8 (Fig. 1) showing numerous synthetic and antithetic coseismic ruptures along the HW and FW of the Principal-CoR (thick red line). (**b)** Rupture-to-rupture distances showing examples of: (i) the most distant FW CoRs and the Principal-CoR (FW-RZW), (ii) the most distant HW CoR and the Principal-CoR (HW-RZW), and (iii) the most distant CoRs at the FW and HW CoRs (Tot-RZW). (**c)** Illustration of RZW calculation (in section view) along topographic profile 1023. (**d)** Along-strike distribution of the RZWs measurements. On the x-axis we report the IDs of the topographic profiles along which the measurements were acquired (topographic profiles are 25 m spaced).

We used the topographic profiles and mapped rupture traces to measure additional fault parameters including the Rupture Zone Width (RZW). The RZW measures the rupture-to-rupture distance between the two most distant CoRs crossed by the topographic profile. Where a main trace is identified, we also measured the HW- and FW-RZW. RZW measurements could be affected by scarp degradation that hides rupture traces. Likely the affect is minimal because only the two most external ruptures are used, the measurement is the rupture-to-rupture distance and the ruptures are clearly identifiable in the DEMs and orthomosaics. We show an example in map view (Figs. 4a, b) and in section view (Fig. 4c) illustrating the RZW measurement and an along-strike plot (Fig. 4d) showing the distribution between HW, FW, and Tot-RZWs.

**Fig. 5:**
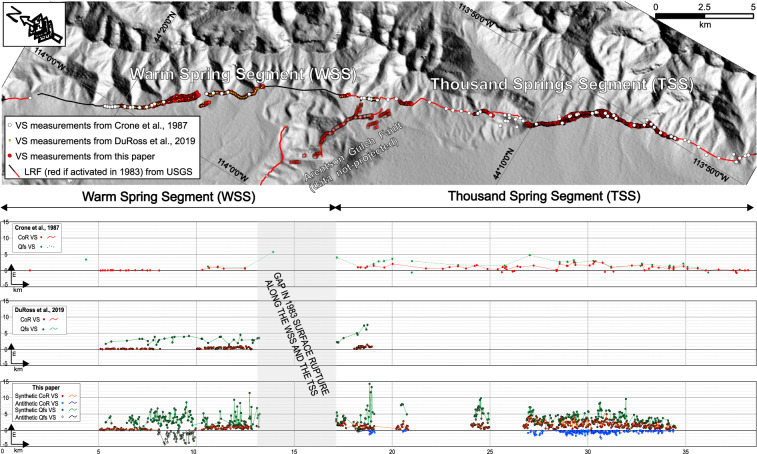
Along-strike distribution of VS measurements acquired along the Warm Springs Segment and along the Thousand Springs Segment from this paper and from previous papers (Crone *et al*.^13^; DuRoss *et al*.^23^). The profile from this work shows separately the sum of the VS measured on synthetic CoRs and Qfs as positive values and the sum of antithetic CoRs and Qfs as negative values.

We calculate the VS from the vertical distance between the intersection of a plane at the fault and lines projected along the HW and FW topographic surfaces. We assume that the surface was continuous prior to their displacement. Consistent with the literature^61^, we calculate the VS instead of the vertical displacement (i.e., throw according to McCalpin^62^) described by McCalpin^62^ as the “vertical distance between intersections of the fault plane, and planes [lines] formed by the displaced original geomorphic surfaces”. Calculating throw would require knowing the CoR’s dip. Using this approach we can make our measurements comparable to those from DuRoss *et al*.^23^ who used a similar methodology to measure VS along the northern 16 km of the 1983Eq and to field-based measurements from Crone *et al*.^13^ ^63,64^. We show along-strike profiles of the VS measurements acquired along the Warm Springs and Thousand Springs Segments in Fig. 5 (and for a better resolution in Supplementary Figure 1) and the distance along-strike of the measurements acquired along the WSS and TSS in the Supplementary Table 1. The fields of Supplementary Table 1 are repeated from the VS dataset (described below). On the profiles (Fig. 5 and Supplementary Figure 1) we plotted separately on the along-strike the sum of the VS measured on synthetic CoRs and Qfs as positive values and the sum of antithetic CoRs and Qfs as negative values. We also report the location of the measurements from Crone *et al*.^13^ and from DuRoss *et al*.^23^ and the along-strike profiles made with their data, as well as a correlation plot comparing a subset of the VS measurements from this paper and from Crone *et al*.^13^, and DuRoss *et al*.^23^ papers (see Supplementary Figure 2 and its description). Unlike DuRoss *et al*.^23^, our topographic profile locations have a fixed spacing over the entire extent of the investigated areas. Following Salisbury *et al*.^63^, the choice not to identify correlative surfaces with the best scarp preservation is likely to decrease subjectivity and biases from selecting only high-quality features. If it is true that subjectivity decreases with this approach, it is also likely that consequently there is a corresponding increase in VS noise. This noise is due to complexities such as vegetation (e.g. bushes and shrubs), surface erosion (e.g. gully erosion), anthropogenic structures (e.g. irrigation channels, excavations, trenches). To minimize these effects, we acquired the measurements by carrying out an assiduous control of the surrounding conditions of the topographic profiles on 3D models, orthomosaics and DEMs. This made it possible to identify, and therefore select, the places of the topography without complexities (creating projection lines on the bare ground, avoiding, for example, bushes or gullies).The 2-m averaging window minimized the impact of the topography. In addition, as described above, a MQR was subjectively assigned to each acquired measurement, which results low on areas of which the geologists observed one or more complexities. Furthermore, we did not acquire VS measurements where the topography was clearly conditioned by anthropogenic structures.

In summary, our measurement database is self-contained and well documented so that other investigators can examine our individual measurements.

## Data Records

We acquired numerical, textual, and graphical datatypes. We have chosen the most appropriate repository for each datatype, whose formats and features we define here.

The data record consists of:

1. High-resolution photogrammetric products in numbered locations in Fig. 1. These were processed from survey campaign photographs using Agisoft Metashape. Metadata are summarized in Table 1. Point clouds are saved in.laz format and the orthomosaics and Digital Elevation Models are saved in GeoTIFF format. Datasets were processed and analyzed in the WGS1984 geographic coordinate system with UTM Zone 12 N projection (EPSG: 32612) and stored in the OpenTopography repository^30^.
2. A shapefile (feature type: polyline) where each line represents a trace of a CoR or a Qfs mapped by the analysis of high-resolution images (example in Figs. 3a and 4a,b). The shapefile keeps in its attribute table: (*i)* an identification number (called “trace ID”) which identifies the trace in a uniquely and which corresponds to the identification number of the first column in Table 2, (*ii)* the field “Type”, a text value to make the categorization of individual traces immediate according to their characteristics, facilitating the use of the database on ArcMap© platforms, (*iii)* a field (called “dip”) indicating the dip direction (~west- or ~east-dipping) to differentiate the synthetic from the antithetic structures and, (*iv)* the OQR (described above). The shapefile is stored in the *Pangaea* repository^34^.
3. A shapefile (feature type: polyline) of the topographic profiles constructed to acquire the VS measurements, stored in the *Pangaea* repository^34^.
4. 1295 topographic profiles figures, saved from the MATLAB analysis, in.pdf format, stored in the *Pangaea* repository^34^.
5. A traces dataset made up of 757 records organized in 21 fields.
6. A topographic profiles dataset made up of 1295 records organized in 23 fields.
7. A measurements dataset made up of 2053 records organized in 16 fields.

The three dataset tables provided in this work (points 5, 6, and 7 above) were uploaded in the *Pangaea* repository^34^ as .TXT files. We have chosen to repeat some initial fields of framing the three datasets to make each of them self-consistent and facilitate their use. Each of the fields have a name and a short name and are uniquely coded in the first row. The fields that make up the three datasets are described below.

### TRACES dataset

Each of the record listed in this dataset reports the trace location, a summary of the measurements acquired on each trace and the geometric characteristics of them. An example of the records is shown in Table 2.

1. **Trace ID** (short name: Tr.ID) number that identified the single trace; this is reported also in the attribute table of the traces shapefile;
2. **Trace Type** (short name: Type): the type of trace between the following three items: Principal Surface Coseismic Rupture (P_CoR), Distributed Surface Coseismic Rupture (D_CoR), Quaternary fault scarp (Qfs);
3. **Fault Name** (short name: Fault): the name of the fault along which the trace has been mapped; (Lost River Fault = LRF, and Lone Pine Fault = LPF)
4. **Fault Segment Name** (short name: Seg.): text indicating the acronym of the fault segment name along which the trace has been mapped; (Challis Segment = CS; Warm Springs Segment = WSS; Thousand Springs Segment = TSS; Mackay Segment = MS; Pass Creek Segment = PCS; Arco Segment = AS. The acronym of the LPF has been repeated in this field as it is not divided into segments: Lone Pine Fault = LPF); (see Table 1).
5. **Area ID** (short name: Area): number that identified the single area where the trace was mapped (Area numbers reported in Fig. 1 and Table 1);
6. **Segment average dip direction** (short name: SDD - expressed in degrees): the direction of the segment dip with respect to the North;
7. **Trace longitude start point** (short name: Lon start): the longitude of the trace start point in decimal degrees (dd.mmmmm) within the Geographic Coordinate System WGS 1984;
8. **Trace latitude start point** (short name: Lat start): the latitude of the trace start point in decimal degrees (dd.mmmmm) within the Geographic Coordinate System WGS 1984;
9. **Trace longitude end point** (short name: Lon end): the longitude of the trace end point in decimal degrees (dd.mmmmm) within the Geographic Coordinate System WGS 1984;
10. **Trace latitude end point** (short name: Lat end): the latitude of the trace end point in decimal degrees (dd.mmmmm) within the Geographic Coordinate System WGS 1984;
11. **Trace Position relatively to the main fault trace** (short name: Pos): text indicating whether the trace is located at the hanging wall (HW) or footwall (FW) of the Principal CoR. When the trace itself represents the Principal CoR, a P (Principal) is indicated. Undefined positions are referred to as NaN;
12. **Synthetic/Antithetic** (short name: Syn/Ant): this field summarizes the information obtainable from the dip direction of each trace, as it categorizes all the traces with the same dip direction of the LRF (synthetic, SW-dipping) and all the traces with dip direction opposite to the LRF (antithetic, NE-dipping).
13. **Trace average strike** (short name: Strike - expressed in degrees): the azimuth angle of a trace with respect to the North;
14. **Trace average dip direction** (short name: Dip dir. - expressed in degrees): the direction of the trace dip with respect to the North;
15. **Trace length** (short name: Length - expressed in meters): the length of a trace automatically measured on ArcMap©;
16. **Outcrop Quality Ranking** (short name: OQR): number between 1 and 4 indicating the quality parameter assigned to each trace based on the clarity of the outcrop observed on the hillshade maps and orthophotos. 1 equals an extremely well visible trace, 4 equals a not clearly visible trace / uncertain / concealed (see the paragraph Methods);
17. **Mean vertical separation** (short name: VS - expressed in centimeters): the average of the VS measurements acquired on the trace of the Qfs, P_CoR or D_CoR, obtained by averaging the values of VS measured;
18. **Standard Deviation** (short name: STD - expressed in centimeters): the amount of variation of the measurements of VS acquired on the trace (Qfs, P_CoR or D_CoR);
19. **Min vertical separation** (short name: Min - expressed in centimeters): the minimum value of VS among the measurements acquired along the trace (Qfs, P_CoR or D_CoR);
20. **Max vertical separation** (short name: Max - expressed in centimeters): the maximum value of VS among the measurements acquired along the trace (Qfs, P_CoR or D_CoR);
21. **Mean vertical separation uncertainty** (short name: Uncert - expressed in centimeters): the average of the uncertainties calculated (as in the Technical Validation section) on the individual measurements acquired along the trace (Qfs, P_CoR or D_CoR).

**Table 2:** Traces dataset, example of 10 records.

Tr. ID	Type	Fault	Seg.	Area	SDD [deg]	Lon start	Lon end	Lat start	Lat end	Pos	Syn /Ant	Strike [deg]	Dip dir. [deg]	Length [m]	OQR	VS [cm]	STD [cm]	Min [cm]	Max [cm]	Uncert [cm]
1	D_CoR	LRF	TSS	9	246	−113.83263	−113.83248	44.11096	44.11072	HW	Syn	159	249	29.2	4	42.9	0	42.9	42.9	15.8
2	D_CoR	LRF	TSS	9	246	−113.83236	−113.83258	44.11026	44.11060	HW	Syn	157	247	43.8	1	105	0	105	105	10.2
3	P_CoR	LRF	TSS	9	246	−113.83243	−113.83111	44.11079	44.10958	P	Syn	132	222	196.4	1	129.1	105.3	29.4	302.4	22.9
4	D_CoR	LRF	TSS	9	246	−113.83190	−113.83147	44.11015	44.1100	NaN	Syn	112	202	38.7	3	105.4	0	105.4	105.4	20.8
5	D_CoR	LRF	TSS	9	246	−113.8321	−113.83144	44.11009	44.10976	HW	Syn	124	214	65.9	1	28.7	6.5	21.3	33.3	19.7
6	P_CoR	LRF	TSS	9	246	−113.83111	−113.83095	44.10947	44.10919	P	Syn	163	253	35.1	1	61.4	61.4	18	104.8	14.4
7	P_CoR	LRF	TSS	9	246	−113.83093	−113.83099	44.10900	44.10918	NaN	Ant	347	77	21.3	1	NaN	NaN	NaN	NaN	NaN
8	D_CoR	LRF	TSS	9	246	−113.83233	−113.83208	44.11013	44.10990	HW	Syn	147	237	34.8	1	35.4	0	35.4	35.4	5.5
9	D_CoR	LRF	TSS	9	246	−113.83138	−113.83167	44.10914	44.10945	HW	Ant	330	60	43.1	2	30.6	0	30.6	30.6	2.7
10	D_CoR	LRF	TSS	9	246	−113.83132	−113.83090	44.10938	44.10863	P	Syn	163	253	94.2	1	31.6	26.8	8.6	66.8	5.6

### Topographic profiles dataset

This dataset reports topographic information, RZW measurements and the cumulated VS of the CoRs and Qfs traces crossed by each topographic profile. An example of the records is shown in Table 3.

1. **Topographic Profile ID** (short name: ID): text indicating an abbreviation with which the topographic profile is uniquely identified, corresponding to a sequential number from North to South. The Topographic profile ID is also present in the shapefile attribute table of the topographic profiles;
2. **Fault name** (short name: Fault): text indicating the name of the fault across which the topographic profile has been traced; (Lost River Fault = LRF, and Lone Pine Fault = LPF);
3. **Fault Segment name** (short name: Seg.): the name of the fault segment where the topographic profile has been traced; (Challis Segment = CS; Warm Springs Segment = WSS; Thousand Springs Segment = TSS; Mackay Segment = MS; Pass Creek Segment = PCS; Arco Segment = AS. The acronym of the LPF has been repeated in this field as it is not divided into segments: Lone Pine Fault = LPF);
4. **Area ID** (short name: Area): number that identified the area where the topographic profile was constructed (area numbers reported in Fig. 1 and Table 1);
5. **Topographic profile longitude start point** (short name: Lon start): the longitude of the topographic profile start point in decimal degrees (dd.mmmmm) within the Geographic Coordinate System WGS 1984;
6. **Topographic profile latitude start point** (short name: Lat start): the latitude of the topographic profile start point in decimal degrees (dd.mmmmm) within the Geographic Coordinate System WGS 1984;
7. **Topographic profile longitude end point** (short name: Lon end): the longitude of the topographic profile end point in decimal degrees (dd.mmmmm) within the Geographic Coordinate System WGS 1984;
8. **Topographic profile latitude end point** (short name: Lat end): the latitude of the topographic profile end point in decimal degrees (dd.mmmmm) within the Geographic Coordinate System WGS 1984;
9. **Average elevation** (short name: Elev – expressed in meters a.s.l.): the topographic profile average elevation above sea level, measured from the DEMs produced for this work;
10. **Variation in topographic elevation** (short name: Δ Elev. - expressed in meters): the variation in meters between the highest point and the lowest point of the topographic elevation along the topographic profile;
11. **Topographic profile length** (short name: Length - expressed in meters): number indicating the length of the topographic profile;
12. **Topographic profile trend** (short name: Trend - expressed in degrees), the azimuth angle of the topographic profile with respect to the North;
13. **Number of measured synthetic 1983 coseismic surface ruptures** (short name: #S_CoRs): number indicating how many CoRs have been measured on the topographic profile;
14. **Synthetic 1983 coseismic surface ruptures vertical separation** (short name: S_VS - expressed in centimeters): the sum of the VS measurements of synthetic CoRs acquired on the topographic profile;
15. **Number of measured antithetic 1983 coseismic surface ruptures** (short name: #A_CoRs): number indicating how many antithetic CoRs have been measured on the topographic profile;
16. **Antithetics 1983 coseismic surface ruptures vertical separation** (short name: A_VS - expressed in centimeters): the sum of the VS measurements of antithetic CoRs acquired on the topographic profile;
17. **Footwall Rupture Zone Width** (short name: FW-RZW - expressed in meters): distance between the Principal CoR and the furthest Distributed CoR at the footwall. NaN values are reported for the topographic profiles not crossing a Principal CoR;
18. **Hanging wall Rupture Zone Width** (short name: HW-RZW - expressed in meters): distance between the Principal CoR and the furthest Distributed CoR at the hanging wall. NaN values are reported for the topographic profiles not crossing a Principal CoR;
19. **Total Rupture Zone Width** (short name: Tot RZW - expressed in meters): distance between the two furthest CoRs along the topographic profile; NaN values are reported for the topographic profiles crossing only one CoR;
20. **Number of measured Synthetic Quaternary fault scarps** (short name: #S_Qfs): number indicating how many synthetic Qfs have been measured on the topographic profile;
21. **Synthetic Quaternary fault scarps vertical separation** (short name: S_Qfs_VS - expressed in centimeters): number indicating the sum of the VS measurements of synthetic Qfs acquired on the topographic profile;
22. **Number of measured antithetic Quaternary fault scarps** (short name: #A_Qfs): number indicating how many antithetic Qfs have been measured on the topographic profile;
23. **Antithetic Quaternary fault scarps vertical separation** (short name: A_Qfs_VS - expressed in centimeters): number indicating the sum of the VS measurements of antithetic Qfs acquired on the topographic profile;

**Table 3:** Topographic profiles dataset, example of 10 records.

ID	Fault	Seg	Area	Lon start	Lat start	Lon end	Lat end	Elev. [m a.s.l.]	Δ Elev [m]	Lenght [m]	Trend [deg]	#S CoRs	S VS [cm]	#A CoRs	A VS [cm]	FW-RZW [m]	HW-RZW [m]	Tot-RZW [m]	#S Qfs	S Qfs VS [cm]	#A Qfs	A Qfs VS [cm]
863	LRF	TSS	8	−113.876655	44.16627	−113.87175	44.169229	2145.6	74.5	511.4	52	2	98	0	NaN	NaN	NaN	126.6	0	NaN	0	NaN
864	LRF	TSS	8	−113.876284	44.16620	−113.87151	44.169081	2147.8	71.1	497.8	52	3	184	0	NaN	NaN	NaN	133.5	0	NaN	0	NaN
865	LRF	TSS	8	−113.875922	44.16612	−113.87122	44.168962	2148.5	62.1	490.2	52	3	144	0	NaN	NaN	NaN	110.2	1	310	0	NaN
866	LRF	TSS	8	−113.875551	44.16605	−113.87097	44.168823	2147.9	53.3	478.2	52	2	317	0	NaN	NaN	NaN	64.3	1	358	0	NaN
867	LRF	TSS	8	−113.875181	44.16598	−113.87071	44.168682	2148.9	49.6	466	52	1	68	0	NaN	54.3	70.9	125.2	1	371	0	NaN
868	LRF	TSS	8	−113.874812	44.16591	−113.87054	44.168492	2151.4	57	445.4	52	2	164	1	−84	41.4	72.7	114	0	NaN	0	NaN
869	LRF	TSS	8	−113.874433	44.16585	−113.87042	44.168273	2152.5	60	418.6	52	4	409	0	NaN	NaN	54.9	54.9	1	550	0	NaN
870	LRF	TSS	8	−113.874079	44.16577	−113.87014	44.16815	2153.6	60.5	411.2	52	3	277	0	NaN	NaN	31.8	31.8	0	NaN	0	NaN
871	LRF	TSS	8	−113.873861	44.16561	−113.86985	44.168031	2152.2	52.9	418.6	52	1	342	0	NaN	NaN	NaN	NaN	1	710	0	NaN
872	LRF	TSS	8	−113.873633	44.16545	−113.86956	44.167913	2150.3	44.1	425.2	52	1	438	0	NaN	NaN	NaN	NaN	1	570	0	NaN

### Measurements dataset

This dataset reports all measurements with location, geometric characteristics, VS and related parameters. An example of the records is shown in Table 4.

1. **Measurement ordinal number** (short name: Ord_No): number with which the measurement is uniquely identified;
2. **Measurement Type** (short name: Type): the object measured between the following three items: Principal Surface Coseismic Rupture (P_CoR), Distributed Surface Coseismic Rupture (D_CoR), Quaternary fault scarp (Qfs);
3. **Latitude** (short name: Lat): the latitude of the point of measurement in decimal degrees (dd.mmmmmm) within the Geographic Coordinate System: WGS 1984;
4. **Longitude** (short name: Lon): the longitude of the point of measurement in decimal degrees (dd.mmmmmm) within the Geographic Coordinate System: WGS 1984;
5. **Elevation** (short name: Elev. – expressed in meters a.s.l.): the altitude above sea level of the point of measurement, extracted from the DEM produced for this work;
6. **Fault name** (short name: Fault): the acronym of the fault along which the measurement was acquired; (Lost River Fault = LRF, and Lone Pine Fault = LPF);
7. **Fault segment name** (short name: Seg.): the acronym of the fault segment along which the measurement was acquired; (Challis Segment = CS; Warm Springs Segment = WSS; Thousand Springs Segment = TSS; Mackay Segment = MS; Pass Creek Segment = PCS; Arco Segment = AS. The acronym of the LPF has been repeated in this field as it is not divided into segments: Lone Pine Fault = LPF);
8. **Area ID** (short name: Area): number that identified the area where the measurement was acquired (Area numbers reported in Fig. 1 and Table 1);
9. **Topographic profile** (short name: T.Pr.): number indicating topographic Profile ID (Topographic profile ID in Table 3) along which the measurement was acquired;
10. **Trace ID** (short name: Tr.ID): number indicating the trace ID (Tr.ID in Table 2) on which the measurement was acquired;
11. **Trace average dip direction** (short name: Dip_Dir. - expressed in degrees): the direction of the trace dip with respect to the North;
12. **Measure position relatively to the fault main trace** (short name: Position): text indicating whether the measurement is located at the hanging wall (HW) or footwall (FW) of the Principal CoR. When the measurement was acquired along a Principal CoR, a P (Principal) is indicated; undefined positions are referred to as NaN;
13. **Synthetic/Antithetic** (short name: Syn/Ant): the LRF is a SW-dipping fault; This field summarizes the information obtainable from the dip direction of each trace, as it categorizes all the traces with the same dip direction of the LRF (synthetic) and all the traces with dip direction opposite to the LRF (antithetic).
14. **Vertical separation** (short name: VS - expressed in centimeters): the VS measurement acquired;
15. **Measure Quality Ranking** (short name: MQR): number between 1 and 4 indicating the quality parameter assigned to each measurement based on three parameters described in section Methods);
16. **Vertical separation uncertainties** (short name: Uncert. - expressed in centimeters): uncertainty of the VS measurement acquired.

**Table 4:** Measurements dataset, example of 10 records.

Ord No	Type	Lat	Lon	Elev. [m a.s.l.]	Fault	Seg.	Area	T.Pr.	Tr.ID	Dip_Dir. [deg]	Pos	Syn/Ant	VS [cm]	MQR	Uncert [cm]
598	D_CoR	44.238016	−113.926449	2245.13	LRF	TSS	5	680	535	216	HW	Syn	120	2	4
599	P_CoR	44.23869	−113.925769	2254.33	LRF	TSS	5	680	529	247	MT	Syn	154	3	9
600	Qfs	44.238707	−113.925752	2254.97	LRF	TSS	5	680	529	247	MT	Syn	259	2	9
601	Qfs	44.246959	−113.917423	2313.14	LRF	TSS	5	680	756	223	FW	Syn	189	2	15
602	D_CoR	44.237853	−113.926227	2245.67	LRF	TSS	5	681	535	216	HW	Syn	117	1	6
603	P_CoR	44.238737	−113.925335	2259.34	LRF	TSS	5	681	529	247	MT	Syn	144	1	7
604	Qfs	44.246849	−113.917149	2321.46	LRF	TSS	5	681	756	223	FW	Syn	348	1	21
605	D_CoR	44.237671	−113.926024	2245.02	LRF	TSS	5	682	535	216	HW	Syn	125	2	6
606	P_CoR	44.238761	−113.924925	2263.09	LRF	TSS	5	682	529	247	MT	Syn	96	1	5
607	Qfs	44.246758	−113.916853	2331.38	LRF	TSS	5	682	756	223	FW	Syn	326	1	22

## Data Statistical Properties

Further demonstration of the value of the data we present here comes from the following statistical analysis. We mapped a total of 757 traces including 662 CoRs generated by the 1983Eq and 95 Qfs.

**Fig. 6:**
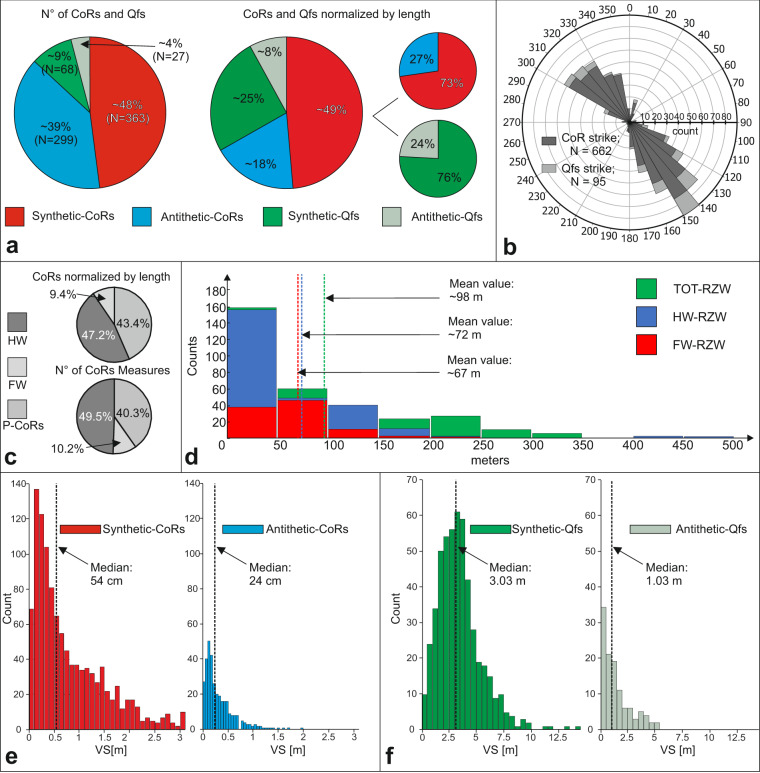
Statistical properties of the data acquired. (**a)** Relative proportions of the 1983 coseismic surface ruptures (CoRs) and of the Quaternary fault scarps (Qfs) mapped; (**b**) rose diagram summarizing the strikes of the fault traces; bin size = 10°; (**c**) relative proportions of the CoRs position between hanging wall (HW), footwall (FW) or main trace (P-CoRs) (upper diagram: number of CoRs mapped normalized by length; lower diagram: number of VS measurements); (**d**) FW, HW and Total-Rupture Zone Width (RZW) frequency distribution; (**e**) CoR VS distribution; (**f**) Qfs VS distribution.

All the mapped traces are divided between synthetic and antithetic, 48% and 39% of the total, respectively, for the CoRs (55% and 45% if considering only CoRs), and 9% and 4% of the total for the Qfs (69% and 31% if considering only Qfs) (Fig. 6a). By normalizing the traces by their length, the synthetic and antithetic traces are respectively 49% and 18% of the total for the CoRs and 25% and 8% of the total for the Qfs. By re-dividing these values for Qfs and CoRs, we obtain a substantial similarity between synthetic, 73% and 76%, and antithetic structures, 27% and 24%, suggesting a recurrence of the subdivision of the surface coseismic deformation for similar events in ~ ¾ on synthetic structures and ~ ¼ on antithetic structures. The total length of the mapped CoRs is ~51 km.

Azimuthal information of the traces indicates the occurrence of a directional peak of strike for synthetic structures at N140°-150° and for antithetic structures a variable strike between N300° and N330° (Fig. 6b).

We characterized the traces of the CoRs and the VS measurements by dividing them into three categories based on their position with respect to the main trace; 43% of the mapped CoRs represent the main trace (Principal-CoRs) while 47% and 9% lie respectively along the HW and the FW. Similarly, 40% of VS measurements represent the trace of the principal CoRs, while 50% and 10% lie respectively along the HW and the FW (Fig. 6c). We characterized the RZW, with widths shown for the HW, FW and total in Fig. 6d. The frequency histogram plot indicate that the FW-RZW averages ~67 m with a median value of ~70 m and a maximum of 236 m. The HW-RZW averages ~72 m with a median value of ~47 m and a maximum of 519 m. Following, for example, Boncio *et al*.^65^, we calculated the Total-RZW by adding the distance between the FW-RZW and the main trace to the distance between HW-RZW and the main trace (rupture-to-rupture distance); where a main trace has not been identified, the Total-RZW refers to the rupture-to-rupture distance between the furthest surface coseismic ruptures along the same topographic profile. For the Total-RZW we obtain an average of ~98 m and a maximum of 519 m. In general, for all ranges of values on the x-axis, the HW-RZW has a higher frequency than the FW-RZW. The values for which the HW-RZW has the same frequency as the TOT-RZW are representative of areas where we did not map CoRs on the FW.

We made 2053 VS measurements. Of these, 1431 are VS measurements of CoRs and 619 are VS measurements of Qfs. The four frequency histogram plots in Figs. 6e and 6f, respectively, show the frequency distribution of these measurements separated by synthetic and antithetic structures.

The VS of the synthetic CoRs is characterized by a sharp peak between 10 and 30 cm and a median value of 54 cm, while the VS of the antithetic CoRs peaks from 10 to 20 cm and a median value of 24 cm (Fig. 6e). The frequency graph of the synthetic Qfs shows a wider distribution of the values between 1.5 and 4.5 m, with a peak between 3 and 3.5 m and a median value of ~3 m; the frequency graph of the antithetic faults shows a peak correspondingt to 50 cm and a median of about 1 m in a decreasing trend up to 5.5 m.

## Technical Validation

**Fig. 7:**
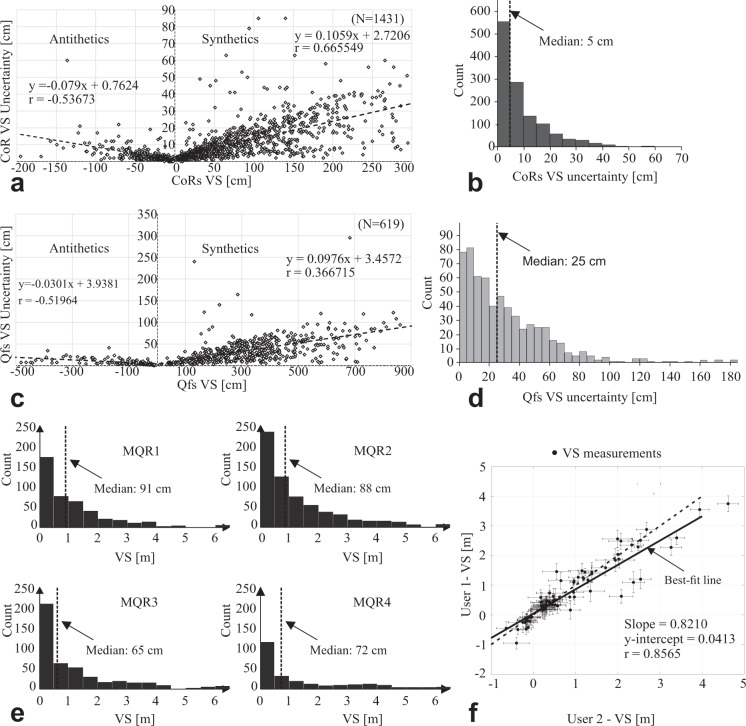
Quality and uncertainty of the measurements. (**a**) Scatter plot of 1983 CoRs VS *versus* uncertainties. Equation of the best-fit line (y) and correlation coefficient (r) are shown on the plot; (**b**) 1983 CoRs VS uncertainty distribution; (**c**) Scatter plot of Qfs VS *versus* uncertainties. Equation of the best-fit line (y) and correlation coefficient (r) are shown on the plot; (**d**) Qfs VS uncertainty distribution; (**e**) VS measurement distribution divided on the basis of the four-class MQR assigned; (**f**) comparison of VS measurements performed by two different operators. The black dashed line represent the ideal best-fit line while solid line represent the real best-fit line. Slope, intercept and correlation coefficient (r) are shown on the plot.

Even with the high-quality of the topographic data, and the efficiency and consistency of the profile analysis tools, the measurements still were made by humans. There are sources of both aleatory and epistemic uncertainty in the VS measurements^63^. While the aleatory uncertainty is considered to be irreducible, inherent and due to chance, the epistemic uncertainty is considered to be reducible, subjective and due to lack of knowledge^66,67^. The sources of these uncertainties are manifold. Scarp changes from erosion and deposition after the rupture induce uncertainty in the reconstruction. During VS measurement, decisions regarding the final geometric model of the area of interest are made based on the scientist’s confidence in interpreting the topographic profile. The points chosen for the regression lines, for example, despite the possibility of being able to control the preservation status of the outcrop thanks to 3D topographic models, DEMs and orthomosaics, can modify the final result in terms of VS. A further source of epistemic uncertainty is due to the choice of the topographic profiles directions. As stated above, the vertical component of displacement is not affected by the variation of the angle of the topographic profile with respect to the strike of the faults. This statement is theoretically true, but it does not take into account a number of complexities that arise from the landform geometry. For example, the footwall and hanging wall that may have dissimilar slopes or slope-facing directions. In choosing the directions of the 1295 topographic profiles interpreted to generate our dataset, we took into account the average strike of the fault in the different areas but, with serial profiles, it is not possible to consider all the innumerable strike changes both along-strike and on CoRs parallel to each other. This source of uncertainty cannot therefore be considered negligible, although, in most cases, it is minimal. Similarly, the fault locations chosen can also vary the final result. For these reasons, the epistemic uncertainty is considered likely to exceed the aleatory uncertainty. With the assignment of the quality parameters made in this work (described above), and with the calculation of a statistical uncertainty (aleatory), we have tried to constrain the values of our data as much as possible. The VS database with uncertainty measures enables end users to decide whether to use values with high-quality ratings only, for example. As discussed in Salisbury *et al.*^63^, the difficulty of correctly interpreting the offset of earthquake ruptures may also depend on the natural variability of the slip along-strike. In numerous previous cases, important variations, even greater than 30%, have been documented within a few units of meters or tens of meters^68,69^. Also in this case, the subjectivity of the scientists plays an important role in acquiring the measurement and in establishing its reliability, avoiding the conditioning of the measurements acquired in the immediate vicinity. To assess subjectivity, two geoscientists experienced in fault scarp studies measured VS. After an initial comparison to standardize the basic scientific knowledge and literature on the LRF area, and to decrease the operator biases, as discussed in Gold *et al*.^70^ and in Scharer *et al*.^71^, and suggested by Salisbury *et al*.^63^, the two operators interpreted the topographic profiles independently, dividing the profiles to be interpreted with even and odd numbers. 10% of the profiles were randomly chosen to be analyzed twice by both geoscientists. As shown in Fig. 7f, ~90 of the ~100 repeated measurements overlap within error, for VS separation, ranging from -50 cm (antithetic) and about 1.5 m (synthetic). Errors on each measurement are calculated by assuming 50 cm error in CoR position.

For quality control, we assigned a quality ranking for each trace during the mapping phase. The ranking corresponds to the evidence of the trace on the hillshade and therefore to the outcrop quality (OQR, described above in the section Methods). Perfectly evident traces were ranked highly (OQR = 1) while poorly evident traces received a low rating (OQR = 4). While measuring VS we reviewed traces mapped on the hillshade on the ArcMap© platform but that were not evident in topographic profiles. In many cases, low ranked traces were eliminated. Following this procedure, we improved the trace and VS dataset quality and decreased uncertainty^63^.

We assigned two independent uncertainties to the vertical separation measurements.

1. A manually assigned while interpreting the topographic profiles. We assigned each VS measurement a rating (MQR, described in the section Methods) based on the confidence accounting for three factors: i) presence of vegetation, ii) angle between the linear surface projections (at the HW and at the FW) and iii) position of the trace. Figure 7e shows the frequency distribution of the VS measurements based on their assigned MQR. For the 2053 VS measurements, we assigned a MQR = 1 (high-quality) to 470 measurements, a MQR = 2 to 780 measurements, a MQR = 3 to 523 measurements and a MQR = 4 (low-quality) to 280 measurements.
2. A quantitative fault VS error (aleatory uncertainty) based on the identified HW surface projection, FW surface projection (see Fig. 3b,c), and fault location. We use a non-weighted linear least-squares inversion to solve for the best-fit line to elevation measurements along a 2 m wide swath along both the HW and FW. Along the FW, the best-fit line ($Foo{t}_{line}$) is

1 $$Foo{t}_{line}={m}_{foot}x+{b}_{foot}$$

Where ${m}_{foot}$is the slope, *x* is the position along the profile, and ${b}_{foot}$ is the y-intercept. Along the HW, the best-fit line ($Hangin{g}_{line}$) is

2 $$Hangin{g}_{line}={m}_{hanging}x+{b}_{hanging}$$

where ${m}_{hanging}$is the slope and ${b}_{hanging}$ is the y-intercept. We perform a coordinate transformation so that the coordinate system origin is at the location of the fault (${x}_{fault}=0$). The fault VS is the difference between $Foo{t}_{line}$ and $Hangin{g}_{line}$ at the location of the fault,

3 $$VS=Foo{t}_{line}-Hangin{g}_{line}={b}_{foot}-{b}_{hanging}$$

We solve for the uncertainty in the VS ($\Delta VS$) using a propagation of uncertainty,

4 $$\Delta {\boldsymbol{VS}}=\sqrt{{\left({m}_{foot}-{m}_{hanging}\right)}^{2}\Delta F{x}^{2}+{\left(\Delta {b}_{foot}\right)}^{2}+{\left(\Delta {b}_{hanging}\right)}^{2}}.$$

We found it reasonable to assume an error in the position of the fault $\left(\Delta Fx\right)$ of 25% of the VS, and not a fixed value.

5 $$\Delta Fx=vertical\,separation/4.$$

Assuming a fixed error $\Delta Fx$would have incorrectly estimated the true VS error. We estimate $\Delta {b}_{foot}$ and $\Delta {b}_{hanging}$ based on the covariance matrix with weights based on the average root-mean-square error of Eqs. 1 and 2. Figures 7a and 7c illustrate the relationship between VS measurements and the calculated uncertainties; measurements of separated synthetic and antithetic CoRs are represented with positive and negative values, respectively. Measurement uncertainty generally increases with VS. The CoRs (Fig. 7a) are clustered with small VS and error while the Qfs (Fig. 7c) are less clustered.

CoRs and Qfs show similar best-fit lines. The frequency histogram plot in Fig. 7b indicates that the CoRs uncertainty have a sharp peak between 0 and 5 cm with a median value of 5 cm with rapidly decreasing distribution with increasing uncertainty value. The frequency histogram plot of the Qfs in Fig. 7d shows a peak between 0 and 10 cm and a median value of 25 cm.

## Usage Notes

An in-depth study of earthquake surface rupture facilitates a better understanding of the controls on rupture processes along the fault zone and over time. This new database contributes towards mitigating earthquake hazard from a better understanding of fault sources and normal surface rupture characteristics. Our fault traces, VS and all the other information described above, can be used in a wide variety of ways in multiple geoscience fields. We provide some key examples below.

The 1983 earthquake ruptures, along the Thousand Springs and the southern Warm Springs segments, develops almost entirely in alluvium and colluvium deposits, close to the contact with bedrock^13^. Our data can serves as critical input for scaling relationships for three-dimensional fracturing processes of a fault that cuts both bedrock and soft soils^72–76^. Furthermore, the integration of our database with lithological and geotechnical data, given the large extent of the mapped area and the heterogeneity of the rock types along the Lost River valley, could be used for microzonation studies for areas adjacent to surface rupturing faults^77–82^, along the LRF, and as example in similar contexts.

Measurements of Rupture Zone Width and trace classification inform studies on hazard on the amplitude of HW and FW surface faulting relative to the principal coseismic surface rupture^65,81,82^.

The mapped traces and the VS measurements integrated with other geometric and kinematic information (such as fault dip and lateral-slip components) indicate the surface slip distribution. Integrating this data with seismological data, seismic lines and well data, researchers can reconstruct the relationship between the deep tectonic structures and their surface manifestation^83–86^.

The generation of profiles of VS along strike using data of the Measurements dataset and Topographic profile dataset can be compared and integrated with prior measurements^13,23^ to gain additional scientific knowledge of the LRF, the seismic behavior of the fault segments, earthquake recurrence times, strain rates and propagation of displacements along strike. Further, these results can be compared with global data^87,88^.

The VS data, the quality parameters (OQR and MQR), and the uncertainties can inform future study of subjectivity in the acquisition of similar types of data^63,89,90^ and the comparison between data collected with our methodology and field collected data, whether collected shortly after the earthquake or several decades later.

The mapped Qfs and the VS data along segments activated and not activated by the 1983Eq are useful for the paleoseismological assessment of characteristics and number of earthquakes released by the LRF and for detailed studies, at the outcrop scale, on fault scarps in extensional contexts in the world^18,54–60,72,91–93^. The topographic profiles and the VS data of the mapped Qfs (not activated by the 1983Eq) may be important because after mapping and measuring them with this reproducible methodology, their effects and shapes will be comparable before and after surface faulting when a new earthquake will ultimately occur. This same process could then be applied in any area with similar characteristics. The traces of the Qfs also provide starting points and locations for further palaeoseismological studies.

The high-resolution DEMs and orthoimagery provide valuable support for paleoseismologic, geomorphological and morphotectonic studies^64,93–100^. They can also be input to study the stress field, as from their interpretation new piercing points may be extrapolated and used as displacement vectors^64,72,87,88,92,101–105^.

This entire effort can contribute to constrain the surface fault trace geometry in the areas where we acquired imagery with detail, helping to implement the reliability of the location of the USGS’s Quaternary faults database^106^.

Information about surface faulting is used for seismic hazard studies in similar tectonic contexts in the world. Comparing of our database with similar databases^25,27,28^ could help define probabilistic estimates to refine scaling laws^72,76,107–109^, and could integrate worldwide databases^29^, improving knowledge of global earthquake properties.

## Supplementary information

Supplementary Figures

Supplementary Table 1

## Data Availability

The MATLAB code developed for this work is available from Zenodo^33^. In addition, we created a small sample dataset that can be with the code as well as a complete guide that illustrates fundamental steps from the preparation of the input data to making the VS measurements. The guide and the example dataset are also hosted in Zenodo^33^.
