# Supplementary material for: High-resolution surface faulting from the 1983 Idaho Lost River Fault Mw 6.9 earthquake and previous events
Source: Sci Data. 2021 Feb 26;8:68. doi: 10.1038/s41597-021-00838-6 (PMC7910603; doi:10.1038/s41597-021-00838-6)
Supplement: Supplementary file 1 — Supplementary Figures [file 41597_2021_838_MOESM1_ESM.pdf]

Supplementary to:

High-resolution surface faulting from the 1983 Idaho Lost River Fault M_w 6.9 earthquake and previous events

Simone Bello[1-2], Chelsea P. Scott[3], Federica Ferrarini[1-2], Francesco Brozzetti[1-2], Tyler Scott[3], Daniele Cirillo[1-2], Rita De Nardis[1-2], J Ramon Arrowsmith[3], Giusy Lavecchia[1-2]

[1]DiSPUTer- Department of Psychological, Humanistic and Territorial Sciences, University G. d'Annunzio Chieti-Pescara, Italy.
[2]CRUST- InterUniversity Center for 3D Seismotectonics with territorial applications, Italy
[3]School of Earth and Space Exploration – Arizona State University, USA

Corresponding author: Simone Bello (simone.bello@unich.it)

Content of this supplementary material:

- **Supplementary Figure 1.** Along-strike distribution of VS measurements;
- **Supplementary Figure 2.** Comparison of the VS measurements from this paper and from Crone et al., 1987 and DuRoss et al., 2019.

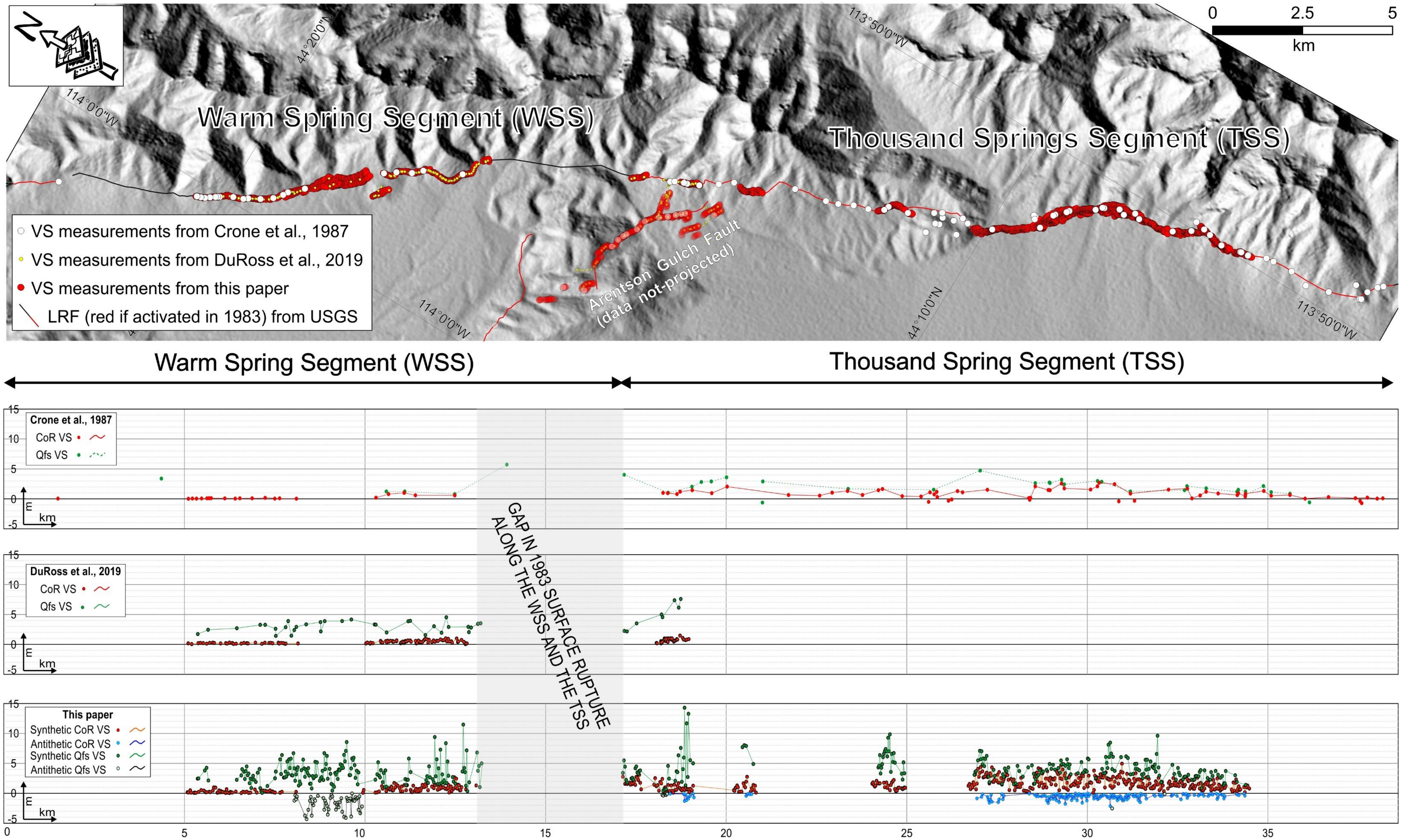

Supplementary Figure 1. Along-strike distribution of VS measurements acquired along the Warm Spring Segment and along the Thousand Springs Segment from this paper and from previous papers (Crone et al., 1987; DuRoss et al., 2019). The profile from this work shows separately the sum of the VS measured on synthetic CoRs and Qfs as positive values and the sum of antithetic CoRs and Qfs as negative values.

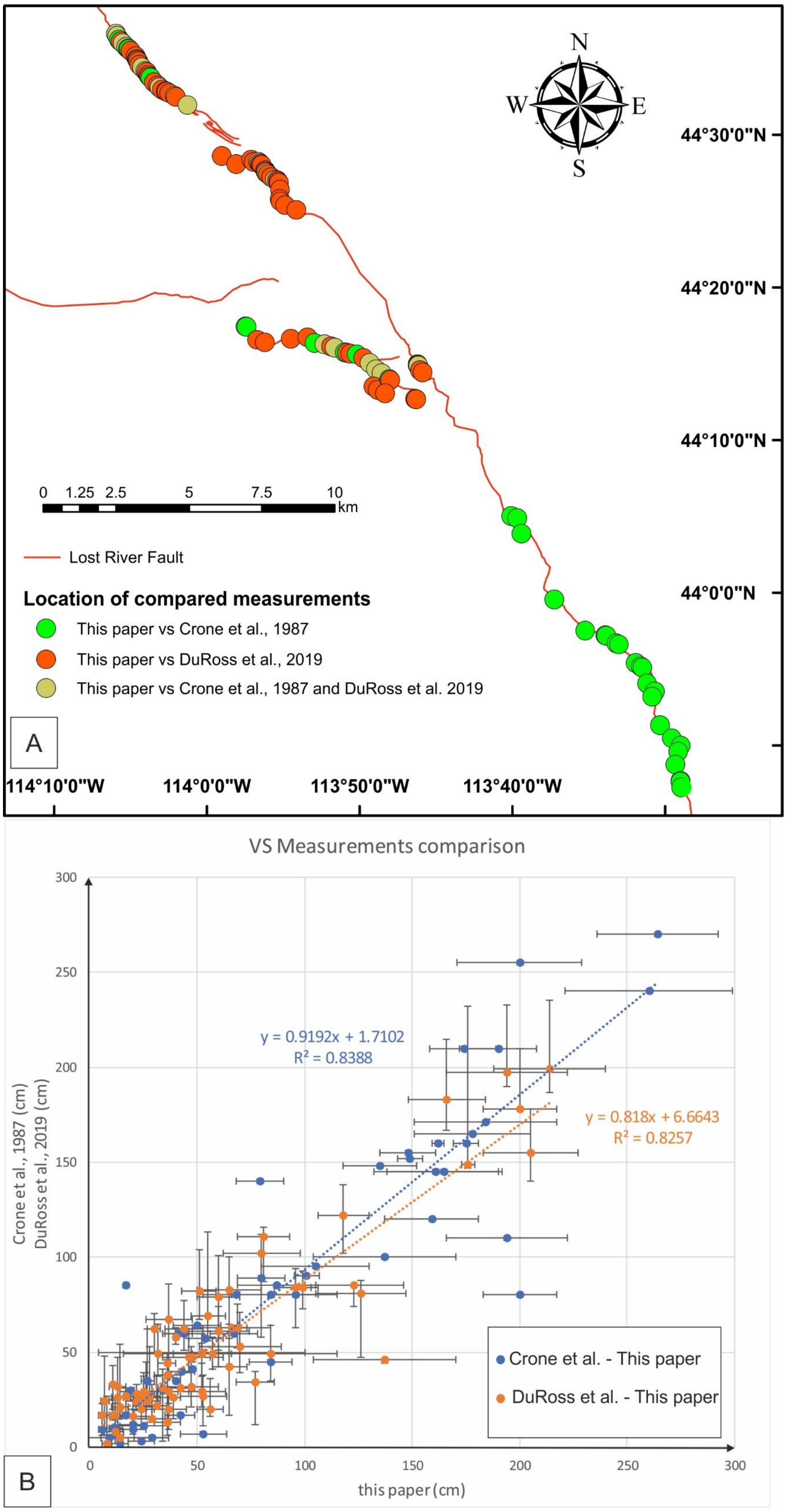

Supplementary Figure 2. Comparison of the VS measurements from this paper and from Crone et al., 1987 and DuRoss et al., 2019. **a)** Map showing the spatial distribution of the measurements we compared. To compare the three datasets, we selected the measurements of this paper based on the measurements of the previous papers, picking the data that were close enough to be unaffected (or minimally affected) by major variations due to possible natural conditions. However, in most cases, the measurements acquired by the three authors are not exactly on the same geographical point. We excluded the data that represented cumulated values (i.e., multiple fault scarps measured together). In the few cases where these data were the only ones available, we compared our and their cumulative values. In total, we compared 125 measurements (53 with Crone et al., 1987, and 72 with DuRoss et al., 2019). **b)** Correlation plot showing DuRoss et al., 2019 (orange points) and Crone et al., 1987 (blue points) *vs* the measurements from this paper. Vertical bars represent the uncertainty reported in DuRoss et al. (2019), while the horizontal ones are the uncertainty related to our measurements. We did not add vertical bars to Crone data because they did not report any uncertainty values in their paper. The linear regression equations and the correlation coefficients, reported in the plot, show that our measurements are comparable with the other two datasets being the slopes of the linear regression of 0.92 (DuRoss) and 0.82 (Crone) with R2 coefficients of about 0.83.
